# Supplementary figures and images for: Regulation of midgut cell proliferation impacts Aedes aegypti susceptibility to dengue virus
Source: PLoS Negl Trop Dis. 2018 May 21;12(5):e0006498. doi: 10.1371/journal.pntd.0006498 (PMC5983868; doi:10.1371/journal.pntd.0006498)

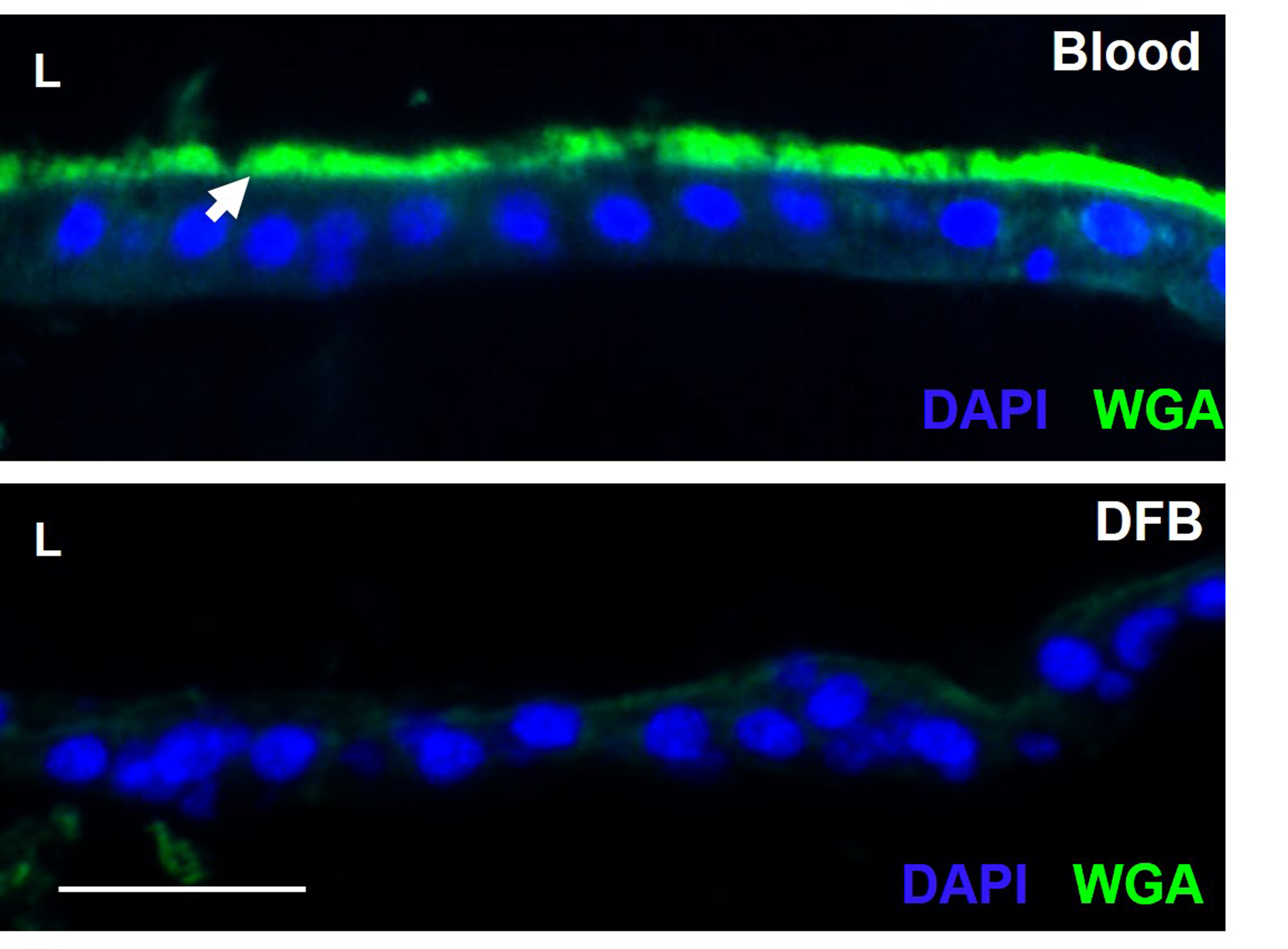

Supplement: S1 Fig — Midguts of females that were fed on blood alone or blood with DFB. Insects were dissected and fixed, and sections 0.14 μm were stained with WGA-FITC (green) and DAPI (blue). The peritrophic matrix is indicated by a white arrow. (L) indicates the luminal side where the PM forms. (TIF) [file pntd.0006498.s001.tif]

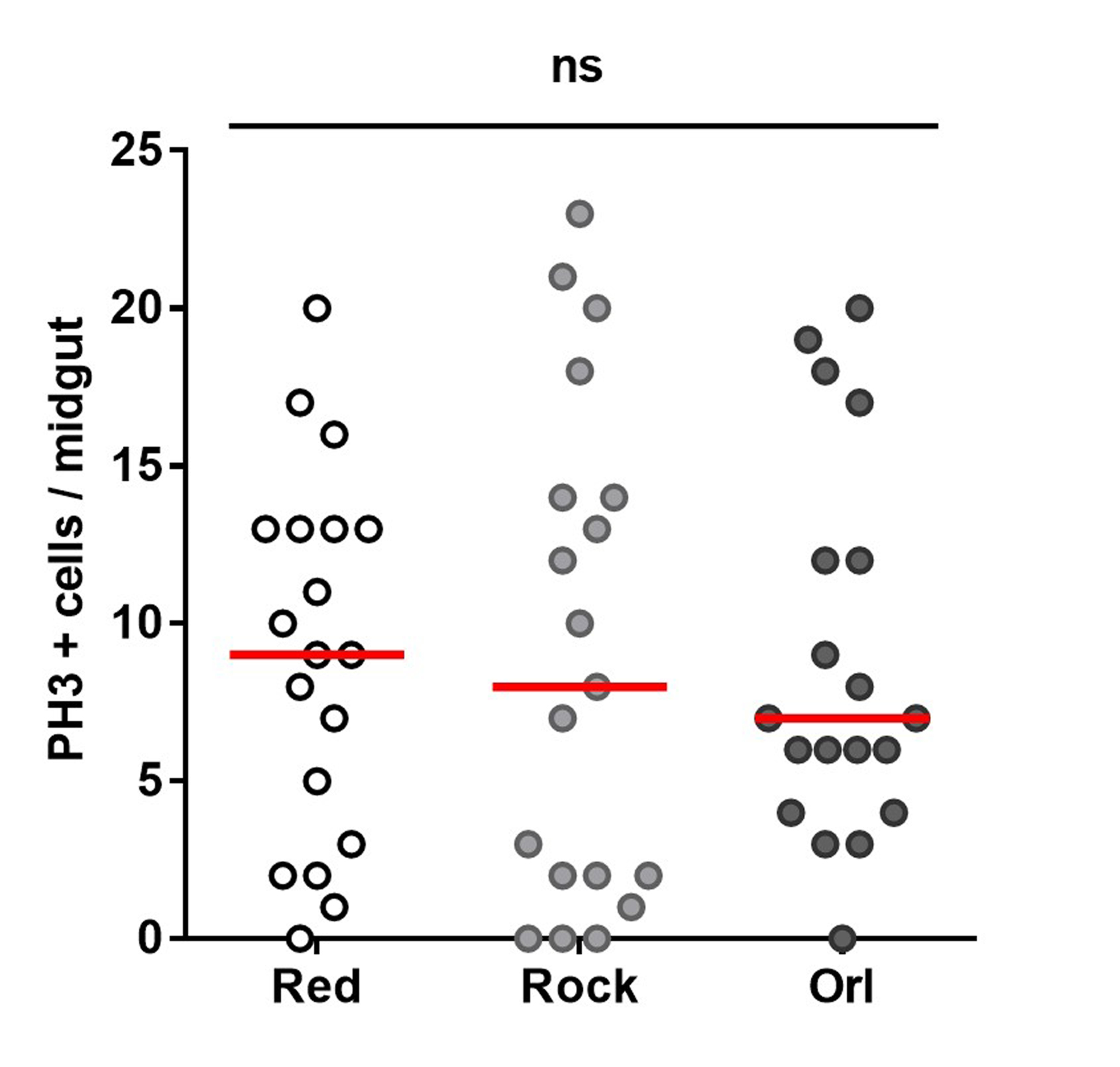

Supplement: S2 Fig — A. aegypti Red-eye, Rockefeller and Orlando strains were collected 5 days after emergence. All PH3 positive cells in the anterior midgut were counted. No significant difference was observed between the groups. Klustal-Wallis test. (TIF) [file pntd.0006498.s002.tif]

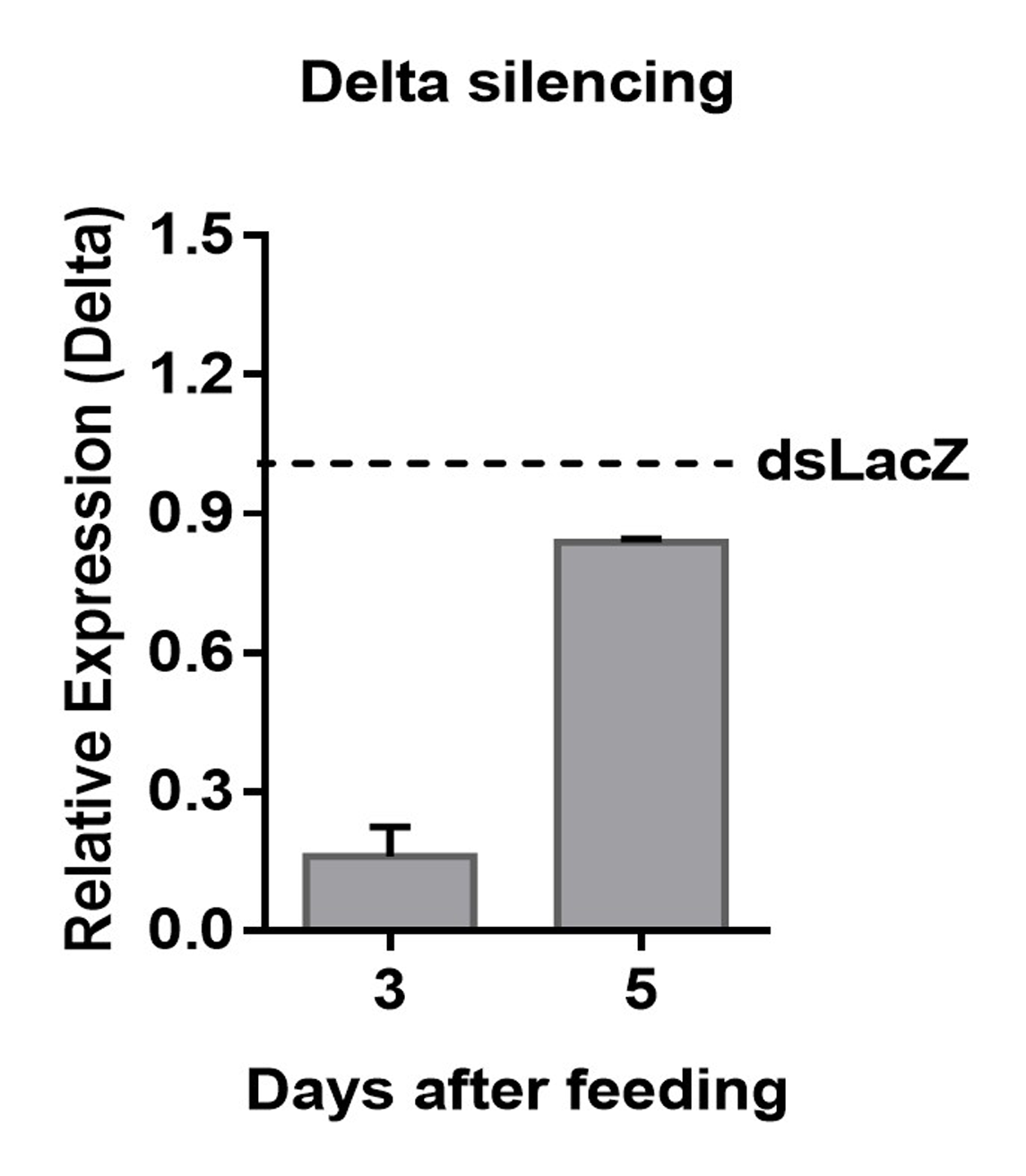

Supplement: S3 Fig — Midguts of females that were injected with dsRNA for the Delta gene were dissected at 3 and 5 days after blood feeding and the RNA extracted for gene expression analysis. dsLacZ was used as experimental control of non- related dsRNA. (TIF) [file pntd.0006498.s003.tif]

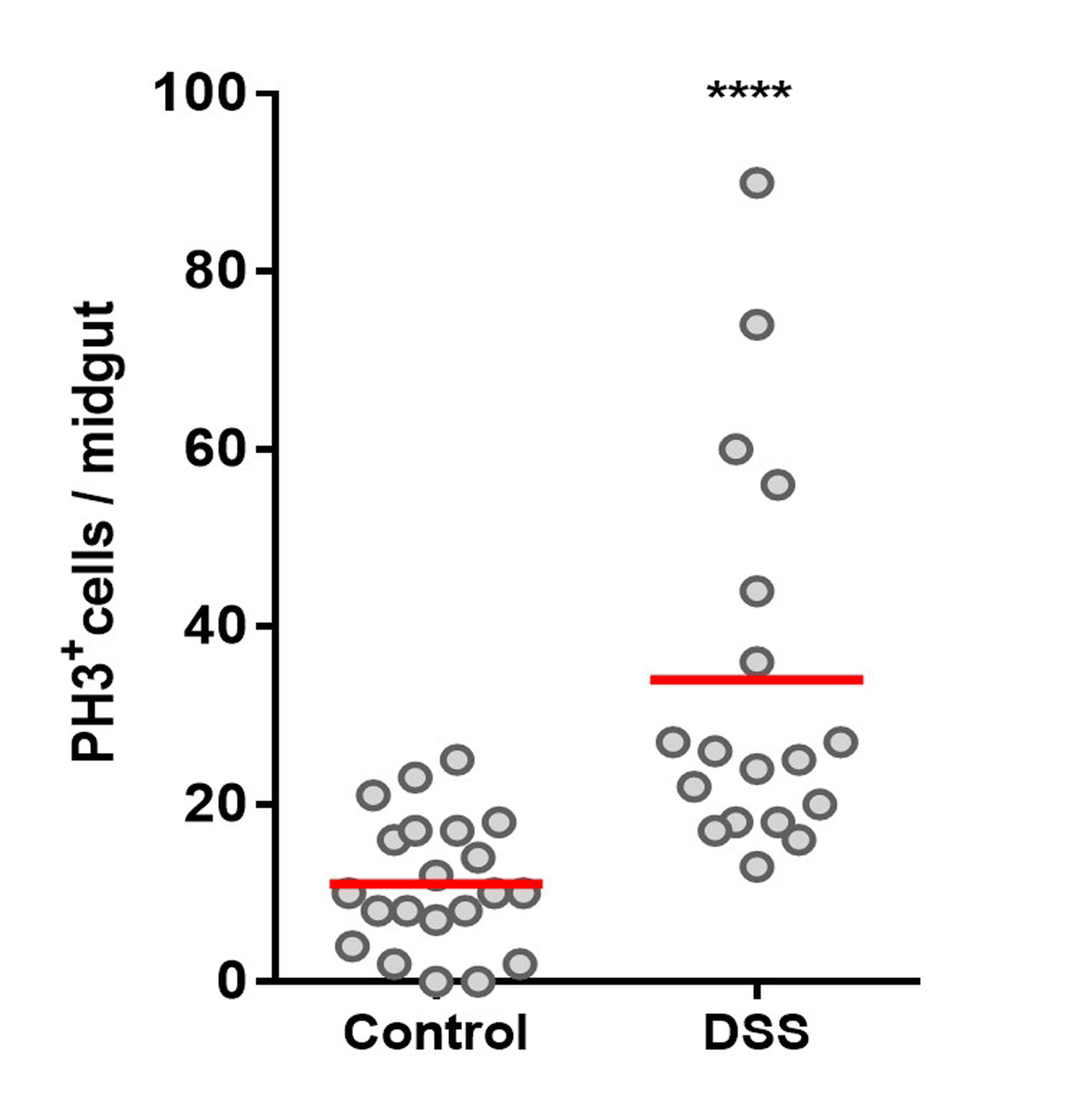

Supplement: S4 Fig — Rockefeller mosquitoes were pre-treated with the tissue-damaging dextran sulfate sodium (DSS) for 3 (three) days and blood fed. After 24 hours, total PH3-positive cells were quantified from midguts of Control (sugar fed) or DSS (1% DSS in the sugar solution) mosquitoes. (TIF) [file pntd.0006498.s004.tif]

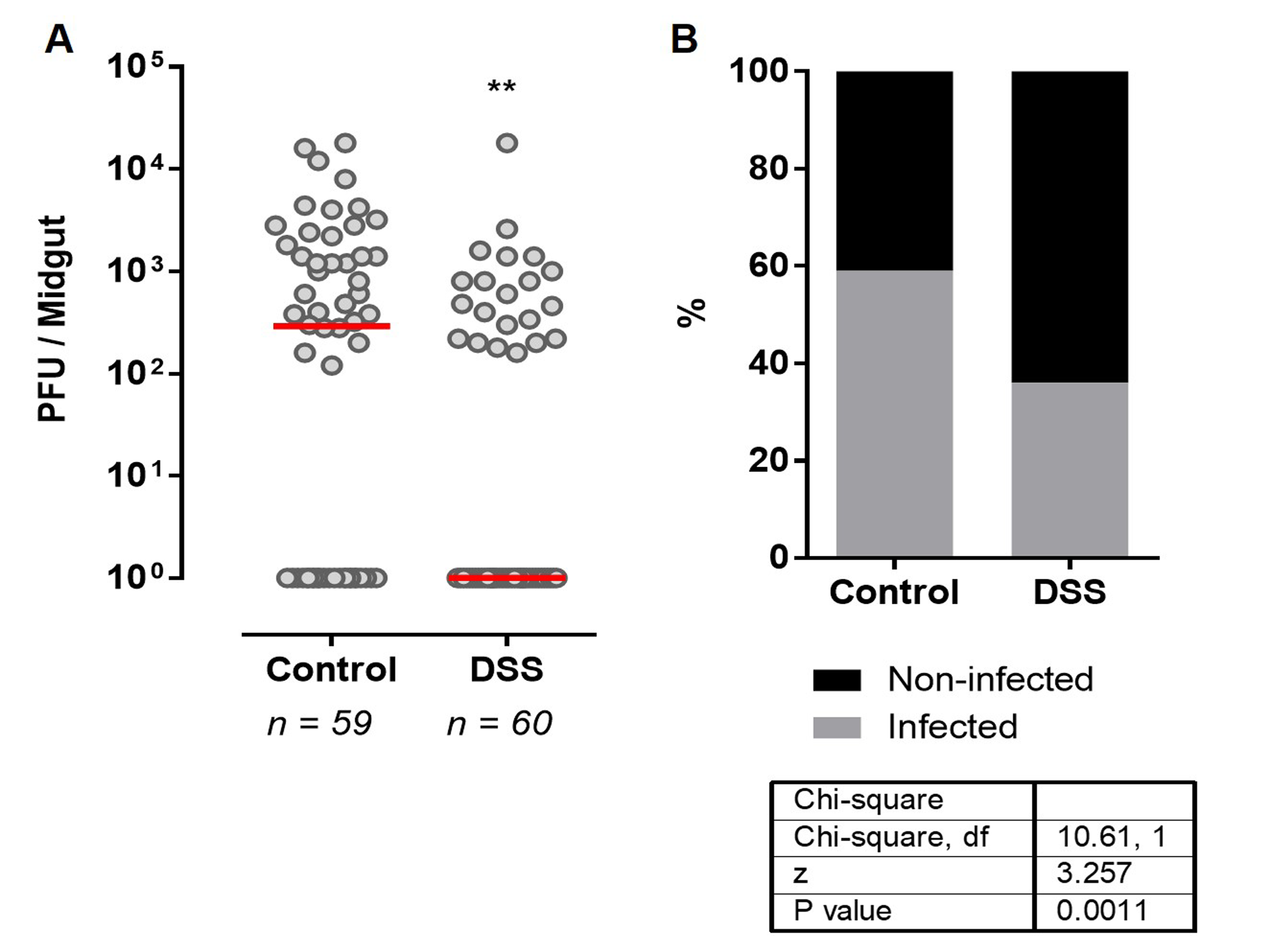

Supplement: S5 Fig — (A) Rockefeller mosquitoes were pre-treated with the tissue-damaging dextran sulfate sodium (DSS) and infected with DENV-4. After 5 days, the midguts were dissected for the plaque assay. (B) The percentage of infected midguts (infection prevalence) was scored from the same set of data as in A. Statistical analyzes used were Mann-Whitney U-tests for infection intensity (A) and chi-square tests for the infection prevalence analysis (B). ** P<0.01. (TIF) [file pntd.0006498.s005.tif]
